# Regional and Local Temporal Trends of *Borrelia burgdorferi* and *Anaplasma* spp. Seroprevalence in Domestic Dogs: Contiguous United States 2013–2019

**DOI:** 10.3389/fvets.2020.561592

**Published:** 2020-10-27

**Authors:** Jenna R. Gettings, Stella C. W. Self, Christopher S. McMahan, D. Andrew Brown, Shila K. Nordone, Michael J. Yabsley

**Affiliations:** ^1^Southeastern Cooperative Wildlife Disease Study, Department of Population Health, College of Veterinary Medicine, University of Georgia, Athens, GA, United States; ^2^Arnold School of Public of Health, University of South Carolina, Columbia, SC, United States; ^3^School of Mathematical and Statistical Sciences, Clemson University, Clemson, SC, United States; ^4^Department of Molecular Biomedical Sciences, Comparative Medicine Institute, College of Veterinary Medicine, North Carolina State University, Raleigh, NC, United States; ^5^Warnell School of Forestry and Natural Resources, University of Georgia, Athens, GA, United States

**Keywords:** *Anaplasma* spp., *Borrelia burgdorferi*, public health, vector-borne, veterinary epidemiology, ticks and tick-borne pathogens

## Abstract

In 2019, in the United States, over 220,000 and 350,000 dogs tested positive for exposure to *Anaplasma* spp. and *Borrelia burgdorferi*, respectively. To evaluate regional and local temporal trends of pathogen exposure we used a Bayesian spatio-temporal binomial regression model, analyzing serologic test results for these pathogens from January 2013 to December 2019. Regional trends were not static over time, but rather increased within and beyond the borders of historically endemic regions. Increased seroprevalence was observed as far as North Carolina and North Dakota for both pathogens. Local trends were estimated to evaluate the heterogeneity of underlying changes. A large cluster of counties with increased *B. burgdorferi* seroprevalence centered around West Virginia, while a similar cluster of counties with increased *Anaplasma* spp. seroprevalence centered around Pennsylvania and extended well into Maine. In the Midwest, only a small number of counties experienced an increase in seroprevalence; instead, most counties had a decrease in seroprevalence for both pathogens. These trends will help guide veterinarians and pet owners in adopting the appropriate preventative care practices for their area. Additionally, *B. burgdorferi* and *A. phagocytophilum* cause disease in humans. Dogs are valuable sentinels for some vector-borne pathogens, and these trends may help public health providers better understand the risk of exposure for humans.

## 1. Introduction

Dogs are frequently exposed to tick-borne pathogens, with *Borrelia burgdorferi*, agent for Lyme disease, and *Anaplasma phagocytophilum*, agent for granulocytic anaplasmosis, being among the most common (1, 2). The primary vectors for *B. burgdorferi* and *A. phagocytophilum* are *Ixodes* spp. *Anaplasma platys* is also implicated in canine anaplasmosis, but is presumed to be transmitted by *Rhipicephalus sanguineus* and causes cyclic thrombocytopenia (3, 4).

The incidence of human granulocytic anaplasmosis and Lyme disease has substantially increased (5, 6) in recent decades, due in part to increased recognition and reporting practices (7). Increasing incidence and geographical distribution (6, 8, 9) are also believed to be related to changes in the distribution and densities of the tick vector *Ixodes* spp., changes in the reservoir host communities, and the interaction between humans and the vector (10–12). The current emergence of *B. burgdorferi* is believed to have started in the 1970s. At that time, previously farmed regions were abandoned as the populace migrated back into cities, and these areas became reforested (10). Reforestation has led to a rise in deer populations, an important host for adult *Ixodes* spp. Further, habitat fragmentation (e.g., suburbanization) has led to an increase in edge habitat which is ideal for small mammals, such as mice, which play a critical role in the enzootic transmission of *A. phagocytophilum* and *B. burgdorferi*. Moreover, the biodiversity of these animal host communities influence the prevalence of infection in ticks (13). Collectively, these changes have lead to an increase in tick populations, and their presence has expanded. For example, counties reporting the presence of *Ixodes* spp. ticks rose from 1,058 in 1998 to 1,531 in 2016 within the contiguous United States (11).

Given the close association between human and canine vector-borne diseases (14–16), the rising number of cases observed in humans and the documented expansion of the vector range raise the question as to whether canine vector-borne disease is increasing as well. Recently, a Bayesian spatio-temporal binomial regression model was developed to describe the temporal trends in these seroprevalence data (17). In that study, the canine seroprevalence of *B. burgdorferi* was found to be increasing. In this study, we explore this further for *B. burgdorferi* using an expanded and more contemporary dataset and apply the model to canine *Anaplasma* spp. seroprevalence in order to identify increases in canine *Anaplasma* spp. or *B. burgdorferi* seroprevalence within the United States.

Recognizing areas of increasing canine seroprevalence is of importance when considering recommendations for preventative care. There are several interventions recommended by veterinarians to reduce the risk of exposure and infection. These include use of acaricides, vaccination against *B. burgdorferi*, regular examination for the presence of ticks, and avoidance of known tick habitats (18). Uncertainty of the level of risk confounds recommendations and compliance with testing and preventative interventions. Clinicians acting on historical experience may fail to initially recognize the increased risk. As such, understanding the dynamic nature of vector-borne disease and change in prevalence is imperative for prevention and control. Furthermore, the pet dogs represented in these data share a peridomestic environment with their human counterparts. The information from these trends can be applied to the One Health approach to the control and prevention of vector-borne disease in humans and animals alike.

## 2. Methods

### 2.1. Data

**Figure 1:**
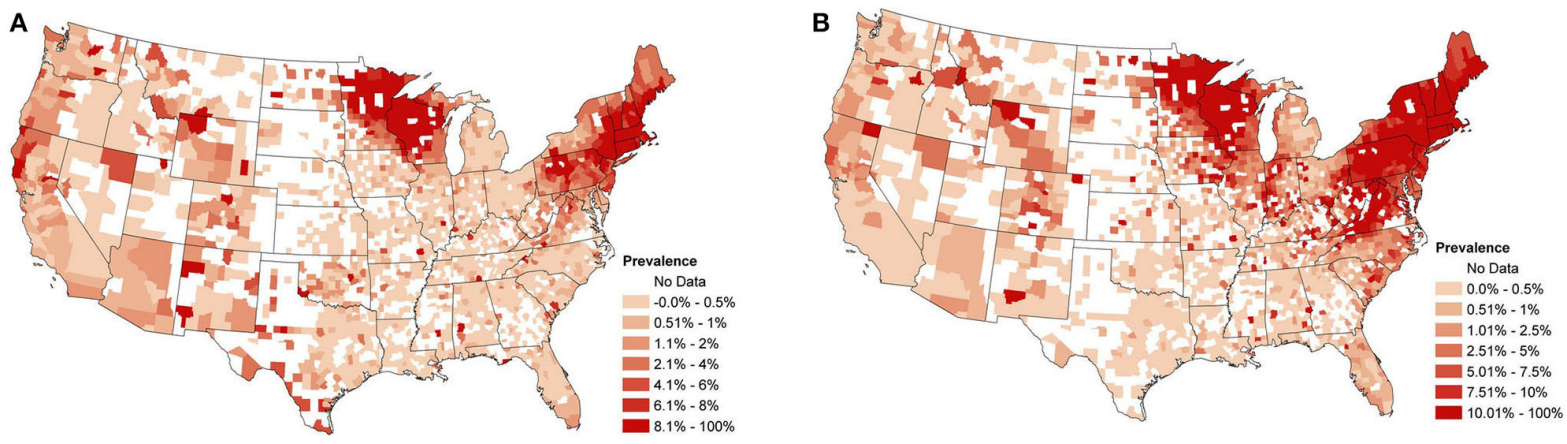
**(A)** Overall observed canine *Anaplasma* spp. seroprevalence from January 2013 to December 2019. Calculated as the proportion of positive test counts among all test counts within a county for the study period. **(B)** Overall observed canine *B. burgdorferi* seroprevalence from January 2013 to December 2019. Calculated as the proportion of positive test counts among all test counts within a county for the study period.

Over 30 million test results for *Anaplasma* spp. and *B. burgdorferi* were collected from point-of-care SNAP®4Dx®Plus (IDEXX Laboratories, Inc. Westbrook, ME) tests, a qualitative modified enzyme-linked immunosorbant assay (ELISA), performed in-clinic and at regional reference laboratories between January 2013 and December 2019 (1). Figures 1A,B depict an aggregation of the *Anaplasma* spp. and *B. burgdorferi* data, respectively, from January 2013 to December 2019. Displayed are the overall raw seroprevalences for each county (the number of seropositive tests divided by the total number of tests reported) over the study period, with white counties being those which did not report any test results. This data presents with strong spatial dependence (*p*-value for Moran's I statistic <0.0001 for both datasets).

The test detects antibodies against *Anaplasma* spp. and *B. burgorferi* as well as *Ehrlichia* spp. (agents of ehrlichiosis) and antigen from *Dirofilaria immitis* (causative agent of canine heartworm disease). It is used commonly by veterinarians throughout the United States for annual screening during wellness examinations or for diagnosis of suspected vector-borne illness. Results were collated automatically by IDEXX Laboratories into a centralized database, from which aggregate data were provided to the investigators at a county and monthly scale. Importantly, the reported county is that of the clinic and no patient histories are known. *Anaplasma* spp. exposure is determined by the presence of antibodies to the major surface protein-2 (p44) of *Anaplasma phagocytophilum* (19). *Anaplasma platys* has been shown to cross-react to p44 (20, 21), and seroconversion for both occurs 10–14 days post-infection (21). For *B. burgdorferi*, the ELISA detects antibodies that are seroreactive to the C6 peptide which is based on invariable region 6, a highly immunogenic and conserved region on the outer membrane protein VlsE of *B. burgdorferi* (22). Seroconversion to C6 occurs after the infection has become disseminated and can occur as early as 3–4 weeks (23).

### 2.2. Model Definition

Regional and local trends were estimated using a Bayesian spatio-temporal binomial regression model. Let *y*_*st*_ and *n*_*st*_ be the number of seropositive tests and total number of tests, respectively, from county *s* in month *t*. We assume

(1) $$\begin{array}{l} y_{st}|n_{st},p_{st}\sim \text{Binomial}\left(n_{st},p_{st}\right), \end{array}$$

where *p*_*st*_ is the unknown seroprevalence in county *s* during month *t*. Thus, we are assuming that given the number of tests and seroprevalence, the number of seropositive tests in county *s* and month *t* follows a binomial distribution with the number of trials equal to the number of tests and the probability of success on each trial equal to the seroprevalence. We model the seroprevalence *p*_*st*_ using a linear predictor η_*st*_:

(2) $$\begin{array}{l} g^{-1}\left(p_{st}\right)=\eta_{st}=\upsilon +\beta \left(s\right)t+\xi_{st}. \end{array}$$

**Figure 2:**
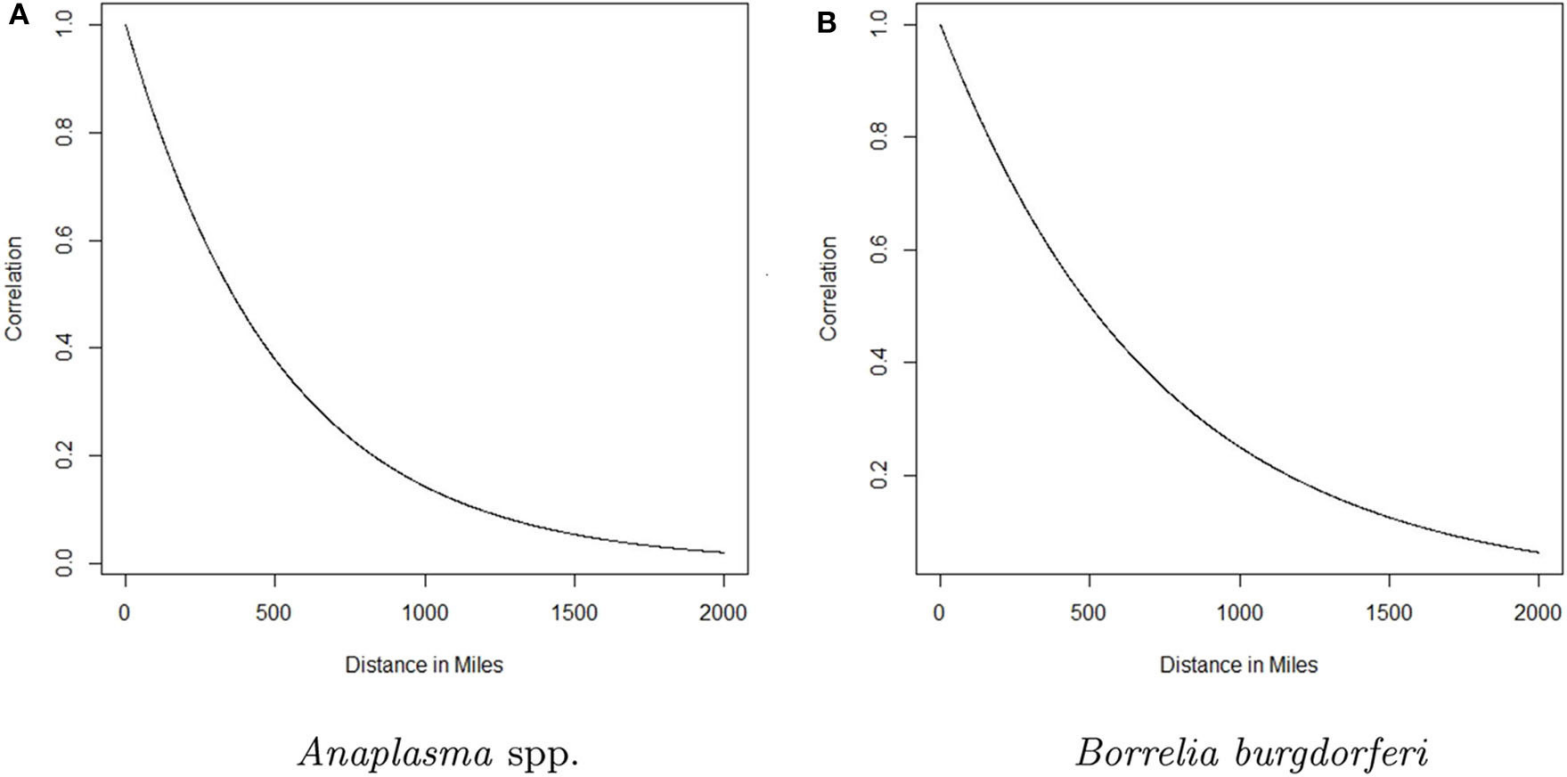
The correlation between the trend parameter for a given county and any other county as a function of distance for *Anaplasma* spp. seroprevalence and *Borrelia burgdorferi* seroprevalence is depicted in **(A,B)**, respectively. The curve represents the decaying influence that any other county has on a given county as the distance between them increases.

Here *g*(·) is the logistic function and ensures *p*_*st*_ will be between 0 and 1, υ is a global intercept term, β(*s*) is the regional trend parameter at county *s*, and ξ_*st*_ is a spatio-temporal random effect included to account for the spatio-temporal dependence in the data. Here *t* denotes time in months, rescaled to be between 0 and 1 for numerical reasons. The β(*s*)'s follow a Gaussian predictive process, which ensures the regional trends change smoothly over space and allows the model to use information from a relatively large surrounding area to estimate the trend at each county. The parent process for the GPP was defined on 100 knot locations, with a mean function **μ**(**ℓ**_*s*_) = 0, and covariance function $\sigma^{2}\rho \left(\ell_{s},\ell_{s^{\prime }};\theta \right)=\sigma^{2}\theta^{d_{s,s^{\prime }}^{2}}$, where **ℓ**_*s*_ and $\ell_{s^{\prime }}$ denote the latitude-longitude of the locations of knot *s* and *s*′, respectively, θ ∈ (0, 1), σ^2^ > 0 and $d_{s,s^{\prime }}$ denotes the euclidean distance between locations *s* and *s*′. Such a covariance function allows the strength of the correlation between two observations to decrease as the distance between them increases (see Figure 2). For additional details, including information regarding the selection of the knot locations, see the Appendix and (17). For more on Gaussian predictive processes, see (24). The data display heavy spatio-temporal dependence. Neglecting to model this dependence can lead to inaccurate estimation and inference. The ξ_*st*_'s are designed to account for this spatio-temporal dependence through a vector autoregession. Specifically, letting $\xi_{t}={\left(\xi_{1t},\xi_{2t},\ldots ,\xi_{St}\right)}^{\prime }$ be the vector of spatio-temporal random effects from all counties at time *t*, we assume

$$\begin{array}{ll} \xi_{1} & \sim N\left(0,\tau^{2}{\left(D-\omega W\right)}^{-1}\right)\\ \xi_{t} & \sim N\left(\zeta \xi_{t-1},\tau^{2}{\left(D-\omega W\right)}^{-1}\right)\text{for}t\geq 2, \end{array}$$

where ζ ∈ (−1, 1) is a temporal correlation parameter, τ^2^ > 0 is a variance parameter, ω ∈ (0, 1) is a propriety parameter included to ensure the invertiblity of the covariance matrix, ***W*** is the *S* × *S* adjacency matrix for the counties (i.e., $W_{ss^{\prime }}=1$ if counties *s* and *s*′ share a border and 0 otherwise), and ***D*** is a diagonal matrix with $D_{ss}=\overset{S}{\underset{j=1}{\sum }}W_{sj}$. The autoregressive structure of our model is similar to that found in (25) and (26), which was based on a generalization of the common conditional autoregressive (CAR) model; for more on CAR models, see (27), (28), or (29). Markov chain Monte Carlo (MCMC) methods were used to fit the model by sampling the unknown parameters from their posterior distributions; with posterior estimation and inference proceeding in the usual manner. Let $\eta_{st}^{\left(g\right)}$ denote the value of the linear predictor calculated using the parameter values from the *g*th MCMC sample. To estimate the local trends for county *s*, we fit the following ordinary least squares model for each value of *g*:

(3) $$\begin{array}{l} \eta_{st}^{\left(g\right)}=\alpha_{0s}^{\left(g\right)}+\alpha_{1s}^{\left(g\right)}t+\epsilon_{st}^{\left(g\right)}\text{for}t=1,\ldots ,T \end{array}$$

where the $\epsilon_{st}^{\left(g\right)}$′s are independent and identically distributed normal random errors. For each county *s*, the $\alpha_{1s}^{\left(g\right)}$′s are a posterior sample of the local trends from county *s*.

In conducting this analysis, a few additional details are of note. First, the data under study presents with county-month pairs for which no tests are reported; e.g., see Figures 1A,B. The spatio-temporal structure in the selected model makes it robust to this sort of missing data, as was demonstrated in (17). Second, to implement the proposed model, a custom MCMC sampling routine was developed and coded in R. This code is available on GitHub (see link at the end of manuscript). Model fitting took approximately 12 h for the *Anaplasma* spp. data and 18 h for the *B. burgdorferi* data on a desktop computer running Windows 10 with an Intel(R) Xeon(R) E-2186G CPU with a 3.80 Ghz processor. For more information on the model, including an explanation of the difference between local and regional trends, details concerning how the model handles missing data, the prior distributions used, and the MCMC sampling procedure, see the Appendix and (17).

### 2.3. Model Selection Procedure

To identify the final model for both datasets, we fit several variants of the model above; e.g., with and without spatio-temporal random effects, with and without spatially varying trends, etc. The deviance information criteria (DIC) was used to select the “best” model from among these candidates. The full model described above had the lowest DIC, and was selected as our final model. To assess the predictive efficacy of these models, the area under the curve (AUC) of a receiver operating characteristic (ROC) curve (30, 31) was computed. That is, the ROC curves were produced for both the *B. burgdorferi* and *Anaplasma* spp. models by plotting the sensitivity (proportion of positive tests correctly predicted) by one minus the specificity (proportion of negative tests correctly predicted) and the area under these curves were computed to determine AUC. In general, an AUC value of 0.70 or greater is considered to be an acceptable model (32).

## 3. Results

### 3.1. Model Accuracy

The AUC for the *B. burgdorferi* trends model was 0.80. The AUC for the *Anaplasma* spp. model was 0.85. Both were >0.70, indicating good predictive performance.

### 3.2. Regional Temporal Trends

The analysis presented in this manuscript provides two perspectives of the changing seroprevalence of *B. burgdorferi* and *Anaplasma* spp. in dogs within the contiguous United States. The regional trend for each county is based on data from a relatively large surrounding area, with the influence of the data from nearby counties diminishing with distance. For a formal depiction of how influence decreases with distance, see Figure 2. Three groups were chosen and designated as high (correlation above 0.75), moderate (correlation between 0.75 and 0.5), and low (correlation <0.5) influence. The distances that correspond to these correlation values based on Figure 2, respectively, are 0–147 miles, 148–357 miles, and >357 miles for *Anaplasma* spp. and 0–207 miles, 208–501 miles, and >501 miles for *B. burgdorferi*. In reality, the influence diminishes in a continuous fashion as distance increases as depicted in Figure 2.

#### 3.2.1. *Anaplasma* spp.

**Figure 3:**
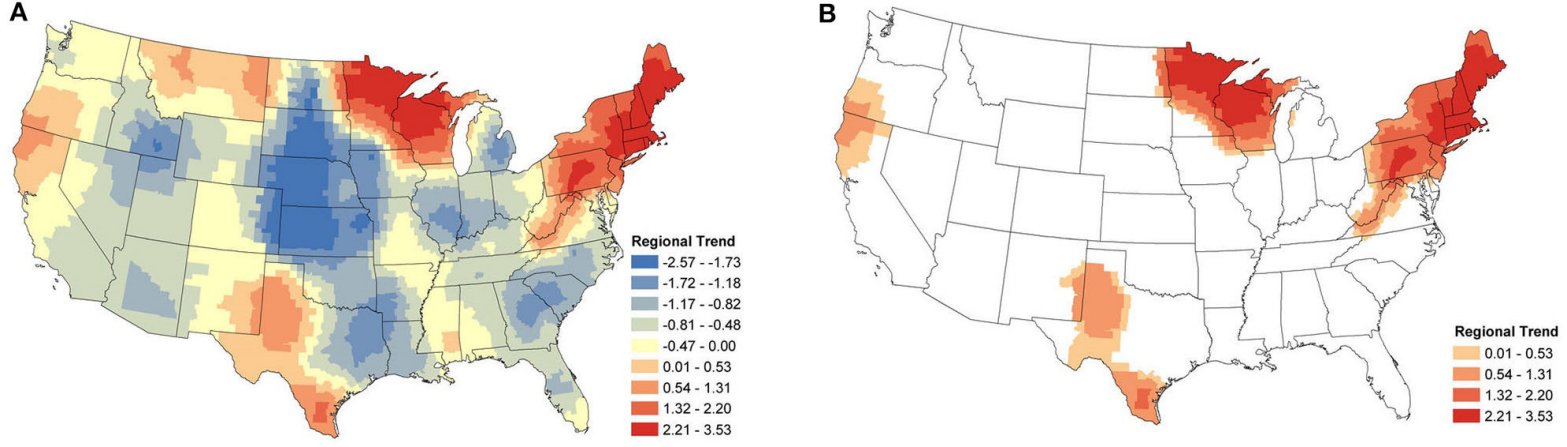
*Anaplasma* spp. Regional Seroprevalence Temporal Trends: **(A)** posterior means of the regional temporal trend parameter β(*s*) from Equation (2); **(B)** counties with a relevant positive trend parameter β(*s*) based on 95% credible intervals. Supplementary Figure 2 depicts the lower and upper bounds of 95% credible intervals for the regional trends from each county.

The regional change in canine *Anaplasma* spp. seroprevalence is shown in Figure 3A. Displayed are the posterior mean values of the temporal trend parameter β(*s*) from equation (2) for each county. Positive values represent an increase in seroprevalence between January 2013 and December 2019, while negative values represent a decrease. Figure 3B shows only the counties deemed to have a relevant positive increase in regional seroprevalence. Herein, the importance of model parameters are determined based on whether their corresponding credible interval captures 0 or not; for further discussion on the interpretation of credible intervals [see (33)]. As is always the case, when simultaneously assessing the relevance of multiple parameters one should be aware of the multiplicity issue (34, 35). The highest positive regional trends are centered in two areas: New England and northern Wisconsin and Minnesota. The surrounding regions experienced smaller changes. In the eastern US, increasing seroprevalence is seen as far as southern Virginia and extends toward the Ohio border. In the Midwest, increasing seroprevalence extends into North Dakota and Iowa. Two foci with smaller positive trends are present in northern California and southern Oregon and in southern and western Texas.

#### 3.2.2. *B. burgdorferi*

**Figure 4:**
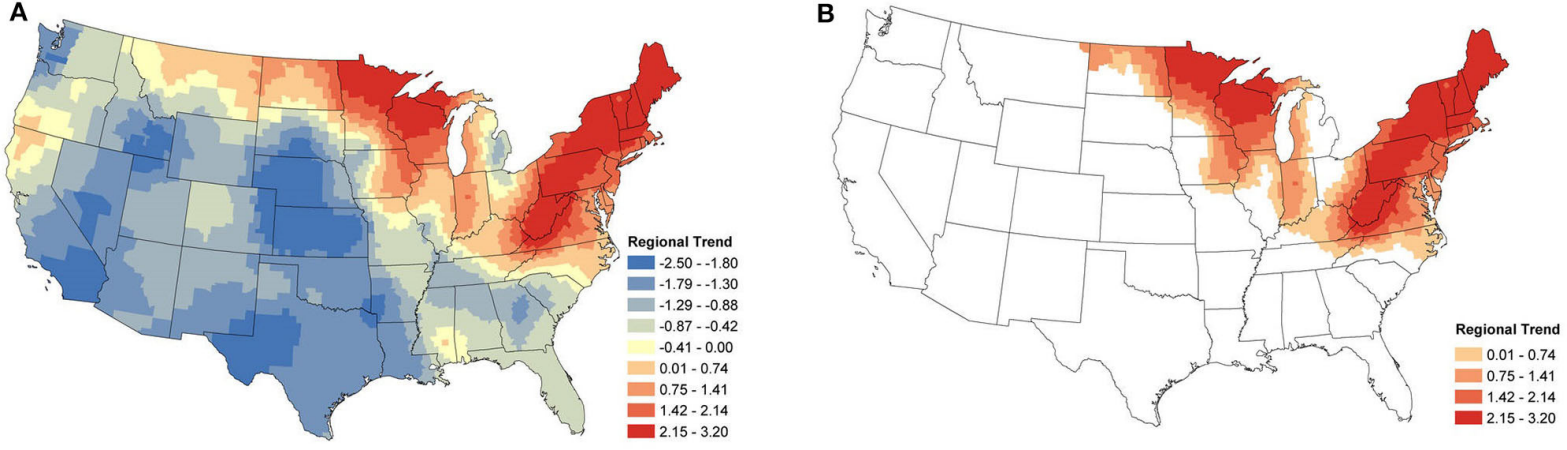
*B. burgdorferi* Regional Seroprevalence Temporal Trends: **(A)** posterior means of the regional temporal trend parameter β(*s*) from Equation (2); **(B)** counties with a relevant trend parameter β(*s*) based on 95% credible intervals. Supplementary Figure 2 depicts the lower and upper bounds of 95% credible intervals for the regional trends from each county.

From Figure 4, we see that the areas with positive regional trends for *B. burgdorferi* seroprevalence were similar to those for *Anaplasma* spp., but with a larger distribution. In the eastern US these trends extend into North Carolina, Tennessee, Kentucky, and Ohio. In the Midwest, these areas extend into Iowa, Illinois, Indiana, Michigan, and North Dakota. No relevant increase in seroprevalence is observed along the West coast (Figure 4B).

The focus of the regional study was positive trends, identifying areas where seroprevalence has increased. However, negative trends can also be identified and for completeness, these maps are available in the Supplementary Material (Supplementary Figure 1). Briefly, areas outside of endemic regions experienced little to no changes. Areas that appear to have large negative trends (e.g., central USA) are areas with very low prevalence (nearly 0%), and consequently, even small changes can create a relatively large trend.

### 3.3. Local Temporal Trends

**Figure 5:**
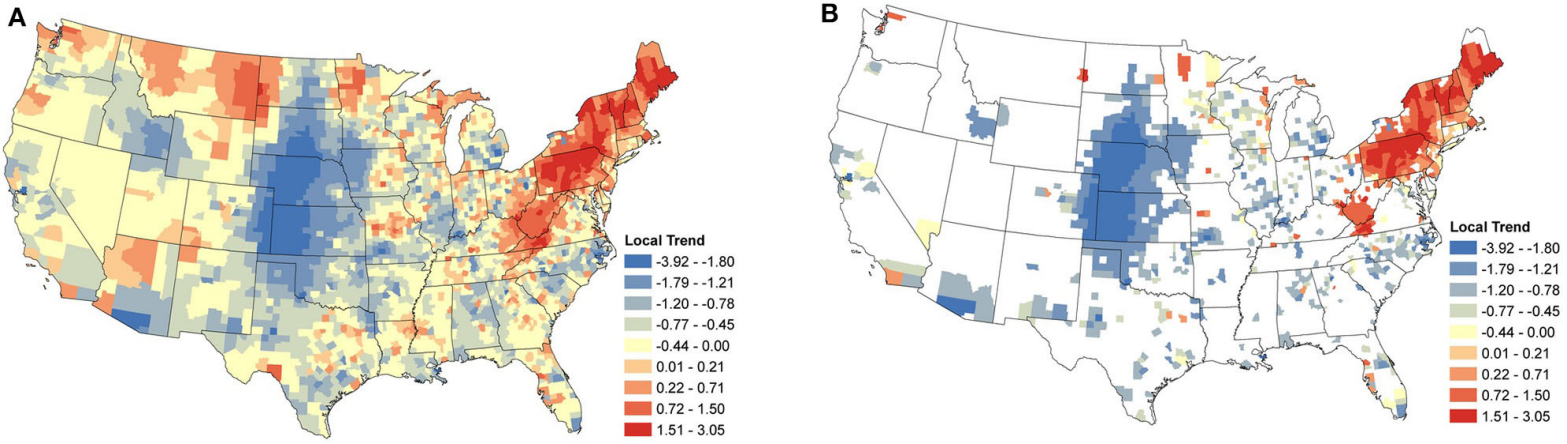
*Anaplasma* spp. Local Seroprevalence Temporal Trends: **(A)** posterior means of the local temporal trend parameter, $\alpha_{1s}^{\left(g\right)}$s, from Equation (3) for all counties; **(B)** posterior means of the positive local temporal trend parameter for counties in which the 95% credible interval did not contain zero. Supplementary Figure 3 depicts the lower and upper bounds of 95% credible intervals for the regional trends from each county.

**Figure 6:**
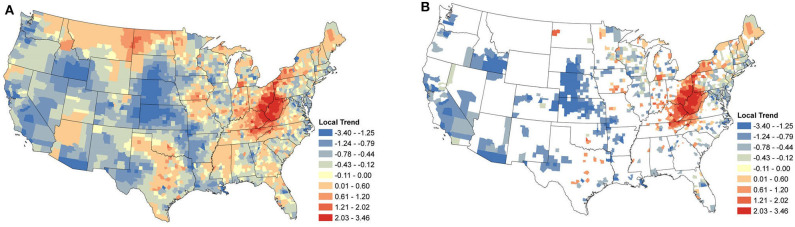
*Borrelia burgdorferi* Local Seroprevalence Temporal Trends: **(A)** posterior means of the local temporal trend parameter, $\alpha_{1s}^{\left(g\right)}$s, from Equation (3) for all counties; **(B)** posterior means of the positive local temporal trend parameter for counties in which the 95% credible interval did not contain zero. Supplementary Figure 3 depicts the lower and upper bounds of 95% credible intervals for the regional trends from each county.

Local temporal trends were also obtained from the same model and reveal the underlying spatial heterogeneity in the changes in seroprevalence (Figures 5A, 6A). These trends are driven predominately by the data from within a single county. As a result, trends can be markedly different, even between neighboring counties. Patterns in the clusters of relevant positive and negative trends are of greatest interest in understanding the local changes.

#### 3.3.1. *Anaplasma* spp.

Focusing on the counties with relevant positive estimates for *Anaplasma* spp. seroprevalence, a large cluster of strongly positive trends were present throughout the Northeast (Figure 5B), with a large area centered within Pennsylvania. Positive counties are seen as far south as Virginia and Kentucky. Perhaps of most interest is the lack of a large cluster of positive trends in Minnesota and Wisconsin, where only a handful of counties are positive. Most of the counties with negative estimates are scattered with little clustering, suggesting local drivers of the trend, but the clusters within North Carolina, Michigan, and much of the Midwest without the intermingling of positive counties does suggest that these areas may have experienced some decrease in seroprevalence in the last several years.

#### 3.3.2. *B. burgdorferi*

Figure 6B shows the relevant local temporal trends for *B. burgdorferi* seroprevalence. The largest cluster of positive trends was centered around the West Virginia and Ohio border, suggesting that this area saw the most change during the study period. This cluster extended into Maine and outward into North Carolina, Kentucky, Tennessee, and Southwestern Ohio. Looking to the Midwest, a loose cluster of positive trends can be seen throughout the Midwest with the larger clusters in Iowa and southwestern Michigan. Negative trends were evident in the majority of the counties along the New England coast extending toward the west. The negative trends also mirrored those of *Anaplasma* spp. in the Midwestern states, particularly Wisconsin and Illinois. Large clusters of negative trends were also scattered throughout the rest of the USA, including the west coast. This was not surprising for the same reason that the regional trends were also negative in many of these areas.

## 4. Discussion

The temporal trends of canine *Anaplasma* spp. and *B. burgdorferi* seroprevalence are described at two different levels of aggregation: regional (multi-state) and local (county). The broader regional trends provide us with an overview of how the prevalence of these pathogens has changed for a general population of dogs over a large spatial area, while the localized county-level trends provide an estimate for the level of risk for populations of dogs within a county. Neither can ascribe a specific risk to an individual dog, but both provide information on how risk is changing over time. It is important to note that these results are relative to the underlying seroprevalence. As such, practitioners are advised to examine seroprevalence at their state and regional level to assess underlying seroprevalence of pathogens and evaluate patient risk (1). For example, there may be regions that do not show an increasing trend; however, the baseline prevalence of a pathogen could be relatively high (but stable).

### 4.1. Regional Temporal Trends

The regional trends analysis show that canine seroprevalence for both *Anaplasma* spp. and *B. burgdorferi* increased broadly in the Northeastern and upper Midwestern states from 2013 to 2019. These changes are likely influenced by the same factors that are driving changes in the geographical distribution and density of *I. scapularis*. The distribution and density of this tick are known to be expanding across a variety of geographical ranges in the United States and Canada (36, 37). This is believed to be predominately driven by climate change that supports vector viability, increases in white-tailed deer population densities, and habitat change such as reforestation and fragmentation (38). In contrast, within the range of *I. pacificus*, little change was seen in *Anaplasma* spp. and *B. burgdorferi* seroprevalence. The only exception was northern California and southern Oregon, where *Anaplasma* spp. seroprevalence increased with relevance. This relative lack of change is supported by the stable population of *I. pacificus*, suggesting that this region is not experiencing the same change as that in the Northeast and Midwest (11).

In most areas, the trends in canine *Anaplasma* spp. and *B. burgdorferi* seroprevalence were similar, with *B. burgdorferi* having a greater geographical distribution. This is consistent with molecular surveys of ticks, molecular testing of *Peromyscus leucopus* (white-footed mouse) reservoirs, and serologic data from dogs. Several molecular studies on *I. scapularis* have shown that the prevalence of *B. burgdorferi* is generally higher than for *A. phagocytophilum* (39–41). Similarly, studies in *P. leucopus* have shown higher prevalences for *B. burgdorferi* compared to *A. phagocytophilum*; for example, prevalence in Wisconsin and Minnesota was 24 vs. 1.7% and 42 vs. 20%, respectively (42, 43). Finally, the seroprevalence of *B. burgdorferi* is much higher in dogs compared with *Anaplasma* spp. (1, 44). In areas extending out from the historically highly endemic regions, the prevalence of *Anaplasma* spp. in *Ixodes* spp. may be below the level of detection and thus impact resolution of change described by this model (45). This, in turn, may explain the disproportionately larger geographical distribution of increased *B. burgdorferi* seroprevalence. However, while lower prevalence of these pathogens in *Ixodes* spp. and important reservoirs helps explain some of the differences we observe in these trends, it would be an over-simplification to not consider the myriad of other factors that impact the ecologies of *Anaplasma* spp. and *B. burgdorferi* (46). In addition, the sensitivity of the currently used *Anaplasma* (SNAP®4Dx®Plus) antigen (APH-1) is lower compared with the *B. burgorferi* C6 peptide which may also explain some differences in prevalence due to missed cases (47–49).

One limitation of our *Anaplasma* spp. data is that the diagnostic test (SNAP®4Dx®Plus) used to measure antibody against *Anaplasma* spp. does not differentiate between *A. phagocytophilum* and *A. platys*. These two pathogens have different expected geographic distributions based on their vectors; however, there are areas that may overlap, particularly in the western United States. *Rhiphicephalus sanguineus* (brown dog tick) (50), the presumed vector of *A. platys*, has a broad geographical distribution that is facilitated in part by its endophilic (indoor dwelling) nature and affinity for domestic dog hosts (51). We are limited in our knowledge of the precise distribution and density of *R. sanguineus* and so the degree to which it overlaps with the ranges of *I. scapularis* and *I. pacificus* is not fully understood. Unlike the potentially broad distribution of *A. platys*, the primary *Anaplasma* sp. expected in dogs is *A. phagocytophilum*, which is restricted to the range of its vectors *Ixodes scapularis* and *I. pacificus*. Thus, *A. phagocytophilum* is believed to account for the trends observed in the northeastern and upper Midwestern regions, and some trends along the western coast.

In this study, areas of increased seroprevalence that may have been impacted by *A. platys* include small regions in California and Oregon and southern Texas. In California and Oregon, increased seroprevalence was observed for *Anaplasma* spp., but not *B. burgdorferi*. This may be due in part to the presence of *A. platys*, but we should also consider the variable diversities of *Ixodes* spp. (52) and their host communities (53) in this region compared to the Northeast and Midwest that may also impact the risk of exposure to dogs. In Texas, Qurollo et al. reported that dogs were equally likely to be exposed to *A. platys* and *A. phagocytophilum* (seroprevalences of 2.0 vs. 2.2%, respectively) (2). This is in contrast to the Northeast where dogs are more frequently exposed to *A. phagocytophilum* (1.5 vs. 13.0%). Given the differences between the vectors of the two *Anaplasma* spp. and the pathogens those vectors carry, future studies would benefit greatly from distinguishing the temporal trends of the two *Anaplasma* spp.

### 4.2. Local Temporal Trends

When the analysis of data shifts from regional trends to local trends, we observe useful information for veterinary practitioners concerned with changes at the county level. At this finer spatial resolution, we are able to see how seroprevalence trends can change over small distances, highlighting the importance of interpreting aggregated data with care. The local trends also appear to highlight areas in which exposure may be newly emergent. Note the trends for *B. burgdorferi* seroprevalence in West Virginia, Ohio, and Kentucky (Figure 6B).

In the Northeast and Middle Atlantic states, we observed a large cluster of counties with increasing local seroprevalence for both *Anaplasma* spp. and *B. burgdorferi*. However, the centers of these clusters were in different locations. The positive trends for *B. burgdorferi* were centered around West Virginia (Figure 6B). This cluster extended in most directions and positive counties were scattered throughout the Northeast, but notably not along the coast. Similar results for canine seroprevalence were obtained by (54) but the main clusters of counties with increasing trends were slightly further north which is likely because their canine serologic data set ended in 2017, 2 years prior to our final year of sampling (54). A recent report of temporal changes in human Lyme disease prevalence mirrors our observations to some extent, although the goals of the two studies differed. Using the first year of detection of human cases and select environmental and demographic factors in their analysis, Bisanzio et al. (55) identified West Virginia as a state with numerous new county reports from 2000 to 2017 and concluded the spread velocity of human Lyme disease estimated by their model was faster in the South. The approach Bisanzio et al. was to analyze the spread of human infections and the likelihood a county would become positive in a given year, but our approach differs in that our model provides data on trends in prevalence for both endemic and non-endemic regions (55). This explains why we had regions of increasing prevalence (e.g., in the Northeast and parts of upper Midwest) that do not show up on maps in Bisanzio et. al as being high risk (because they were already endemic). Thus, differences in the two analyses are likely attributable to four main factors; (1) modeling difference (two step diffusion model vs. a spatio-temporal binomial regression model with spatially varying trend parameters), (2) population under study (dog vs. human), (3) goals of study (predicting first case vs. identifying trends), and (4) the time range over which the study was conducted.

In our analysis, the cluster of counties with increasing *Anaplasma* spp. seroprevalence was centered around Pennsylvania and extended northward through Maine. Similar increasing trends from this same region were reported between 2010 and 2017 by (54). Differences in the prevalence of infection of *B. burgdorferi* and *Anaplasma* spp. in *Ixodes* spp. in these two regions might explain some of this difference, but the prevalence of infection of *I. scapularis* in West Virginia is not known at this time. There is evidence of the expanding range of *Ixodes* spp., with *B. burgdorferi* (8, 9, 45, 56), but as discussed with respect to regional trends, *Anaplasma* spp. may be lagging due to limitations in detection of accurate pathogen prevalence or other factors (45).

Local trends have direct application to veterinary medical decisions. The spatial difference in the temporal trends of *B. burgdorferi* and *Anaplasma* spp. pathogens may be related to differences in preventative practices. Duration of endemicity, awareness of acaricide products, socioeconomic factors, and client-based education vary substantially between endemic and non-endemic regions of the country, thereby affecting the frequency of preventative use (57). In addition, there are currently no protective vaccines on the market for *Anaplasma* spp. in dogs, and few available acaricides on the market currently repel ticks (58). Most acaricides rely on transfer of the drug during the tick bite to kill the tick within hours. This is protective against *B. burgdorferi* which requires a prolonged attachment time (59), but may not protect against pathogens, such as *Anaplasma* spp., that may transmit in a shorter time period (60). As a result, routine practices of preventative care in historically endemic regions may not fully protect against *Anaplasma* spp., and as a result, yield an increase in seroprevalence.

The evidence of increasing exposure to the tick-borne pathogens from this analysis is notable for several reasons. First, increasing seroprevalence suggests that utilization and compliance with recommended year-round use of preventative measures continues to be inadequate in some areas (57), particularly in established endemic regions and neighboring areas. These observations reinforce the concept that veterinarians and pet owners within these regions should recognize the persistent and growing risk of exposure, and implement appropriate preventative measures. Second, even in the presence of acaricides, prompt removal of ticks is strongly recommended to prevent pathogen transmission. Third, given the dynamic nature of tick-borne diseases, veterinarians practicing in regions proximate to endemic areas should adjust screening and preventative care protocols accordingly. Similarly, emphasis should be placed on vaccinating dogs at risk for Lyme disease prior to exposure (61), and the aforementioned areas of increasing seroprevalence provide veterinary practitioners with evidence-based recommendations for use of Lyme disease vaccines against emerging disease. Even in areas where the trends were not increasing, it is imperative that veterinarians and pet owners recognize that dogs are still at risk of exposure, particularly in endemic regions, and that preventative measures and testing should not be discontinued.

Supporting the evidence that dogs can act as sentinels in human vector-borne disease (15, 16), is the similarity in temporal trends between this study and incidence rates of human cases reported by the Centers for Disease Control and Prevention (CDC) and other researchers. Specifically, in regions with strong positive trends in canine *Anaplasma* spp. seroprevalence (i.e., Pennsylvania and northward, Figure 5), positive trends were observed during the same time period in the reported incidence-rate of human anaplasmosis (62, 63). Similar trends have been noted for human *B. burgdorferi* cases in Virginia and West Virginia (8, 64).

Although the focus of this study was identification of locations where the seroprevalence of *B. burgdorferi* and *Anaplasma* spp. was increasing, we noted several areas of that had decreases in seroprevalences. There was a remarkable cluster of counties that had a decrease in *B. burgdorferi* within the northeastern US, predominately along the Atlantic coast. This occurred for *Anaplasma* spp. seroprevalence to a lesser extent. The upper Midwest experienced very little increase, which was surprising given the historical endemicity. Instead, most counties experienced a stable or decrease in seroprevalence for both pathogens. However, similar results were obtained by Dewage et al. who analyzed canine serologic data collected during an earlier time period (2010–2017) (54). Finally, these changes are supported by trends that are observed in CDC-reported human cases of Lyme disease and anaplasmosis, lending further support to the use of dogs as sentinels for these, and possibly other, vector-borne pathogens (15, 16). Recently, several states within the Northeast have reported decreases in the number of human cases reported annually (65). In Wisconsin and Minnesota, trends of human incidence appeared to be stable during this study period (66). It is important to note that these are short term trends, and the long-term implications of these trends are unknown at this time. It is possible that public education and use of preventative practices in these endemic areas may be reducing the risk of exposure and thus reducing the incidence of infection (67). However, knowledge and use of these practices vary within and outside endemic areas (68, 69).

This study focused on two important pathogens associated with *Ixodes scapularis, B. burgdorferi*, and *Anaplasma* spp., which have large amounts of exposure data available through veterinary testing; however, this tick species is also a known vector for several human and zoonotic pathogens including *Babesia microti, Borrelia miyamotoi, Ehrlichia muris* subsp. *eauclarensis*, Powassan encephalitis virus (70), and the recently discovered *B. mayonii* (71). As we point out above, the seroprevalence of *B. burgdorferi* is, in part, a sentinel for the changing population of *Ixodes* spp. and, as a result, should compel both human and veterinary medical practitioners to be cognizant of the potential changes in incidence and spatial distribution of all pathogens carried by *Ixodes* spp. (15, 16). The known ranges for many of these pathogens, e.g., *E. muris eauclarensis* (72) and *B. mayonii* (73), are currently restricted to a small region, so future research is needed to determine what ecological factors drive the presence and distribution of these pathogens and whether there are any correlations with other pathogens transmitted by *Ixodes* spp.

Finally, there are limitations to this analysis. The temporal trends presented do not explicitly show spatial spread of *B. burgdorferi* or *Anaplasma* spp. seroprevalence over the study time period and the results here should not be interpreted as spatial change. The data are from a population of dogs under the care of a veterinarian, and so it is reasonable to assume that these dogs are more likely to be well-cared for and more likely to be provided preventative and medical care. As a result, these results may not reflect changes in higher risk populations of dogs (e.g., shelter and rescue dogs). The trends presented here reflect not only the change in the distribution and prevalence of the pathogens, but also changes in testing and preventative practices, both of which should be considered when interpreting these results. Finally, our data do not include the Canadian territories and provinces. However, there is no physical barrier between the northern US and southern Canadian border to prevent the movement of ticks and *B. burgdorferi* reservoir hosts. There is a growing recognition of canine Lyme disease in Canada along with increased geographic distribution and density of *Ixodes scapularis*, increased numbers of *B. burgdorferi*-infected ticks, increased human cases (74) and increased seroprevalence in dogs (75, 76). Canadian veterinarians and human healthcare providers should take the same precautions as those in the USA practicing in these transitional zones.

## 5. Conclusion

The results of this analysis support the increase in seroprevalence within currently recognized high incidence regions of *B. burgdorferi* and *Anaplasma* spp. in the United States and in the regions immediately surrounding those high incidence areas. Veterinarians and pet owners should take the appropriate precautions to prevent exposure to dogs. Although this analysis does not identify specific risk factors associated with the increasing seroprevalence, there are preventative measures veterinarians and pet owners can take to reduce the risk of exposure and infection. Year-round tick preventative is recommended, particularly in areas where ticks are active into the fall and early spring. Similarities between the trends in exposure to vector-borne pathogens in both the canine and human populations support the use of canine data when estimating the risk of exposure to humans and should be considered when developing predictive models for either population.

## Data Availability Statement

The data presented in this study can be found online. The Borrelia data can be found at https://capcvet.org/maps/#2020/all/lyme-disease/dog/united-states/ while the Anaplasma data can be found at https://capcvet.org/maps/#2020/all/anaplasmosis/dog/united-states/.

## Author Contributions

SS, CM, and DB conceptualized and developed the model. SS wrote the methods. JG, SN, and MY contributed to the interpretation the results and writing of the manuscript. JG, SS, CM, DB, SN, and MY reviewed and edited the manuscript. All authors read and approved the final manuscript.

## Conflict of Interest

The authors declare that the research was conducted in the absence of any commercial or financial relationships that could be construed as a potential conflict of interest.
