# Supplementary material for: Regional and Local Temporal Trends of Borrelia burgdorferi and Anaplasma spp. Seroprevalence in Domestic Dogs: Contiguous United States 2013–2019
Source: Front Vet Sci. 2020 Oct 27;7:561592. doi: 10.3389/fvets.2020.561592 (PMC7653440; doi:10.3389/fvets.2020.561592)
Supplement: Supplementary file 1 [file Data_Sheet_1.PDF]

Supplementary Material for "*Borrelia burgdorferi* and *Anaplasma* spp. seroprevalence in domestic dogs: contiguous United States 2013-2019"

Web Appendix: Additional Details Regarding Model Specification

This appendix provides additional details regarding the model outlined in Section 2 of the manuscript. The methodology was originally developed in Self et al. (2018), and a complete description of the methodology, model fitting procedure, and related simulation studies may be found in that work. For the purposes of convenience and completeness, we here provide additional details regarding the specific form of the model outlined in Section 2 of the manuscript.

1. Modeling Methods

Recall that y_{st} denotes the number of cases (e.g., positive tests) observed in n_{st} tests taken in county s at time t, for $s = 1, \ldots, S$ and $t = 1, \ldots, T$. Set $\boldsymbol{Y}_s = (y_{s1}, \ldots, y_{sT})'$, $\boldsymbol{Y} = (\boldsymbol{Y}'_1, \ldots, \boldsymbol{Y}'_S)' \in \mathbb{R}^{ST}$, $\boldsymbol{n}_s = (n_{s1}, \ldots, n_{sT})'$, and $\boldsymbol{n} = (\boldsymbol{n}'_1, \ldots, \boldsymbol{n}'_S)' \in \mathbb{N}^{ST}$. Following (Self et al., 2018), let Z_{stq} and X_{stp}, for $q = 1, \ldots, Q$ and $p = 1, \ldots, P$, denote covariates associated with location s at time t. The Z_{stq} are covariates whose effects are constant over the study area, while X_{stp} are covariates whose associated effects vary by region. In our model, the set of covariates whose effects are constant over space consists only of a global intercept term (i.e., $Q = 1$ and $Z_{st1} = 1$ for all s and t). The set of covariates whose effects vary by region consists only of the spatially varying regional trend (i.e. $P = 1$ and $X_{st1} = t/T$ for all s and

t). We assume that $y_{st}|n_{st}, p_{st} \sim \text{Binomial}(n_{st}, p_{st})$, where

$$\eta_{st} := g^{-1}(p_{st}) = \boldsymbol{Z}'_{st}\boldsymbol{\delta} + \boldsymbol{X}'_{st}\boldsymbol{\beta}(\boldsymbol{\ell}_s) + \xi_{st}; \quad s = 1, \ldots, S;\ t = 1, \ldots, T. \tag{1}$$

In the above expression, $g : \mathbb{R} \to (0, 1)$ is the logistic link function relating the linear predictor η_{st} to the prevalence p_{st}, $\boldsymbol{Z}_{st} = (1, Z_{st1}, \ldots, Z_{stQ})' \in \mathbb{R}^{Q+1}$, $\boldsymbol{X}_{st} = (X_{st1}, \ldots, X_{stP})' \in \mathbb{R}^P$, $\boldsymbol{\delta} = (\delta_0, \ldots, \delta_Q)'$ are global regression coefficients, $\boldsymbol{\beta}(\cdot) = (\beta_1(\cdot), \ldots, \beta_P(\cdot))'$ are spatially varying regression coefficients, $\boldsymbol{\ell}_s = (\ell_{s1}, \ell_{s2})'$ is a vector of spatial coordinates (e.g., latitude and longitude) that identifies the centroid of region s, and ξ_{st} is a spatio-temporal random effect. In the manuscript, we use $\beta(s)$ to denote $\beta(\boldsymbol{\ell}_s)$. Gaussian predictive processes (GPPs) are used to model the $\beta(\cdot)$ parameters.

A GPP employs a "parent" Gaussian process on a set of "knots" placed throughout the study area and interpolates to points of interest via kriging. Let $\{\boldsymbol{\ell}_1^*, \ldots, \boldsymbol{\ell}_{S_p^*}^*\}$ denote a set of "knot" locations with $S_p^* \ll S$. Define $\boldsymbol{\beta}_p^* = (\beta_p(\boldsymbol{\ell}_1^*), \ldots, \beta_p(\boldsymbol{\ell}_{S_p^*}^*))'$ and note that $\boldsymbol{\beta}_p^*|\sigma_p^2, \boldsymbol{\theta}_p \overset{ind}{\sim} \text{N}(\boldsymbol{0}, \boldsymbol{C}_p^*)$, for all p, where $\boldsymbol{C}_p^* = \sigma_p^2 \boldsymbol{R}_p^*$ and $(\boldsymbol{R}_p^*)_{ss'} = \rho_p(\boldsymbol{\ell}_s^*, \boldsymbol{\ell}_{s'}^*; \boldsymbol{\theta}_p)$. The GPP replaces $\boldsymbol{\beta}_p$ with $\tilde{\boldsymbol{\beta}}_p := E(\boldsymbol{\beta}_p|\boldsymbol{\beta}_p^*; \boldsymbol{\theta}_p) = \tilde{\mathbf{R}}_p^*(\mathbf{R}_p^*)^{-1}\boldsymbol{\beta}_p^*$, where $\tilde{\mathbf{R}}_p^*$ is an $S \times S_p^*$ matrix whose (s, s')th element is $\rho_p(\boldsymbol{\ell}_s, \boldsymbol{\ell}_{s'}^*; \boldsymbol{\theta}_p)$. For our model, we took $\rho_p(\boldsymbol{\ell}_s, \boldsymbol{\ell}_{s'}^*; \boldsymbol{\theta}_p) = \theta_1^{d_{s,s'}^2}$, where $\theta_1 \in (0, 1)$ and $d_{s,s'}$ denotes the euclidean distance between locations s and s'. We selected 100 knot locations via K-means clustering with S_p^* clusters; i.e., using K-means clustering, the S counties are partitioned into S_p^* clusters based on their locations $\boldsymbol{\ell}_s$. The knot locations are taken as the centroids of the S_p^* clusters. Web Figure 6 displays the knot locations used in our analysis.

The likelihood is

$$f(\boldsymbol{Y}|\boldsymbol{\eta}) \propto \prod_{t=1}^{T}\prod_{s=1}^{S} g(\eta_{st})^{Y_{st}}\{1 - g(\eta_{st})\}^{n_{st}-Y_{st}}. \tag{2}$$

where $\boldsymbol{\eta} = (\eta_{11}, \ldots, \eta_{1T}, \eta_{21}, \ldots, \eta_{ST})'$. The data augmentation approach of Polson et al. (2013) is used to facilitate the MCMC sampling routine. This approach allows us to express

(2) as

$$f(\boldsymbol{Y}|\boldsymbol{\eta}) \propto \prod_{t=1}^{T}\prod_{s=1}^{S} \exp(\kappa_{st}\eta_{st}) \int_0^\infty \exp(-\psi_{st}\eta_{st}^2/2) p(\psi_{st}|n_{st},0) d\psi_{st}$$
$$\propto \prod_{t=1}^{T}\prod_{s=1}^{S} \int_0^\infty f_{Y,\psi}(Y_{st},\psi_{st} \mid \eta_{st}) d\psi_{st},$$

where $p(\cdot \mid b, 0)$ is the probability density function of a Pólya-Gamma random variable with parameters b and 0, $\kappa_{st} = Y_{st} - n_{st}/2$ and $f_{Y,\psi}$ is the joint density of (Y_{st}, ψ_{st}). Treating the ψ_{st} as latent random variables to be sampled via MCMC, we obtain

$$f_{\boldsymbol{Y},\boldsymbol{\psi}}(\mathbf{Y}, \boldsymbol{\psi} \mid \boldsymbol{\eta}) \propto \exp(-\boldsymbol{\eta}'\boldsymbol{D}_{\boldsymbol{\psi}}\boldsymbol{\eta}/2 + \boldsymbol{\kappa}'\boldsymbol{\eta}) \prod_{t=1}^{T}\prod_{s=1}^{S} p(\psi_{st}|n_{st},0),$$

where $\boldsymbol{\psi} = (\psi_{11}, \ldots, \psi_{1T}, \psi_{21}, \ldots, \psi_{ST})'$, $\boldsymbol{D}_{\boldsymbol{\psi}} = \text{diag}(\boldsymbol{\psi})$, and $\boldsymbol{\kappa} = (\kappa_{11}, \ldots, \kappa_{1T}, \kappa_{21}, \ldots, \kappa_{ST})'$. Since data are not reported at all county-month pairs, let $\mathcal{R}$ be the set of all ordered pairs (s,t) for which tests are observed. The augmented likelihood is

$$f(\mathbf{Y}(\mathcal{R}), \boldsymbol{\psi}(\mathcal{R}) \mid \boldsymbol{\eta}(\mathcal{R})) \propto \exp(-\boldsymbol{\eta}(\mathcal{R})'\boldsymbol{D}_{\boldsymbol{\psi}(\mathcal{R})}\boldsymbol{\eta}(\mathcal{R})/2 + \boldsymbol{\kappa}(\mathcal{R})'\boldsymbol{\eta}(\mathcal{R})) \prod_{(s,t)\in\mathcal{R}} p(\psi_{st}|n_{st},0),$$

where $\boldsymbol{\eta}(\mathcal{R}) = \boldsymbol{Z}(\mathcal{R})\boldsymbol{\delta} + \boldsymbol{X}(\mathcal{R})\widetilde{\boldsymbol{b}} + \boldsymbol{I}(\mathcal{R})\boldsymbol{\xi}$ and the convention that $\boldsymbol{A}(\mathcal{R})$ is the matrix formed by retaining the rows of $\boldsymbol{A}$ whose indices are in $\mathcal{R}$ is used. Here, $\boldsymbol{Z} = (\boldsymbol{Z}_1', \ldots, \boldsymbol{Z}_S')' \in \mathbb{R}^{ST\times(Q+1)}$ with $\boldsymbol{Z}_s = (\boldsymbol{Z}_{s1}, \ldots, \boldsymbol{Z}_{sT})'$. Similarly, $\boldsymbol{X} = \bigoplus_{s=1}^{S} \boldsymbol{X}_s \in \mathbb{R}^{ST\times SP}$ with $\boldsymbol{X}_s = (\boldsymbol{X}_{s1}, \ldots, \boldsymbol{X}_{sT})'$, $\boldsymbol{I}$ is the identity matrix, and $\widetilde{\boldsymbol{b}} = (\widetilde{\boldsymbol{\beta}}'(\boldsymbol{\ell}_1), \ldots, \widetilde{\boldsymbol{\beta}}'(\boldsymbol{\ell}_S))' \in \mathbb{R}^{SP}$. Since $\boldsymbol{\xi} \in \mathbb{R}^{ST}$ is the vector of spatial random effects over *all* locations within the study region for all time points, the full conditional for $\boldsymbol{\xi}$ is well-defined provided that the prior on $\boldsymbol{\xi}$ is proper. This joint density representation permits the imputation of any missing effects via posterior realizations.

The prior distributions for all other parameters are given below:

$$
\begin{aligned}
\sigma_p^2 &\overset{indep.}{\sim} \text{Inverse Gamma}(2, 2), \quad p = 1, \ldots, P; \\
\theta_p &\overset{i.i.d.}{\sim} \text{Uniform}(0, 1), \quad p = 1, \ldots, P; \\
\boldsymbol{\delta} &\sim \text{N}(\mathbf{0}, 1000\boldsymbol{I}), \quad \sigma_\delta^2 > 0; \\
\tau^2 &\sim \text{Inverse Gamma}(2, 2) \\
\omega &\sim \text{Beta}(900, 100) \\
\zeta &\sim \text{Truncated-Normal}(0, 10, -1, 1),
\end{aligned}
\tag{3}
$$

where $\boldsymbol{I}$ denotes a $Q \times Q$ identity matrix.

Web figures 4 and 5 displays the prior distribution (red) and posterior MCMC sample from each model fit for the hyperparameters.

2. Web Figures

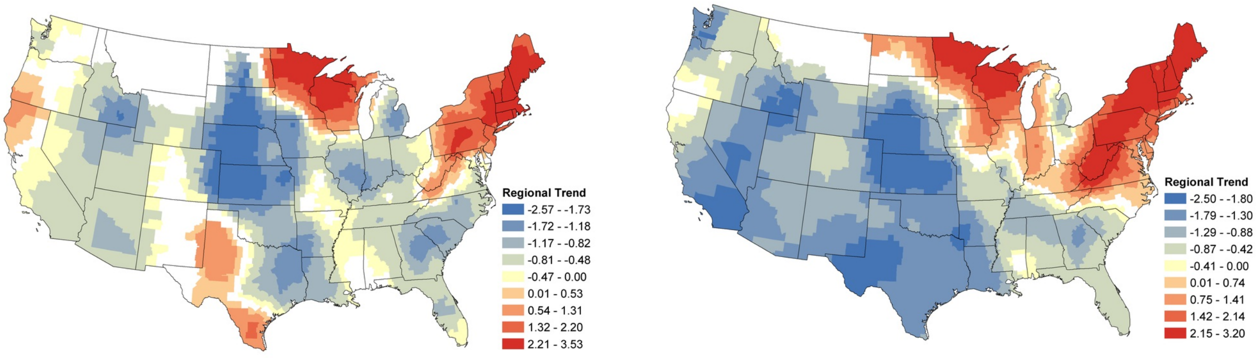

(A) (B)

Web Figure 1: Counties for which the 95% credible interval for the regional trends for *Anaplasma* spp (A) and *B. burgdorferi* (B) does not contain 0.

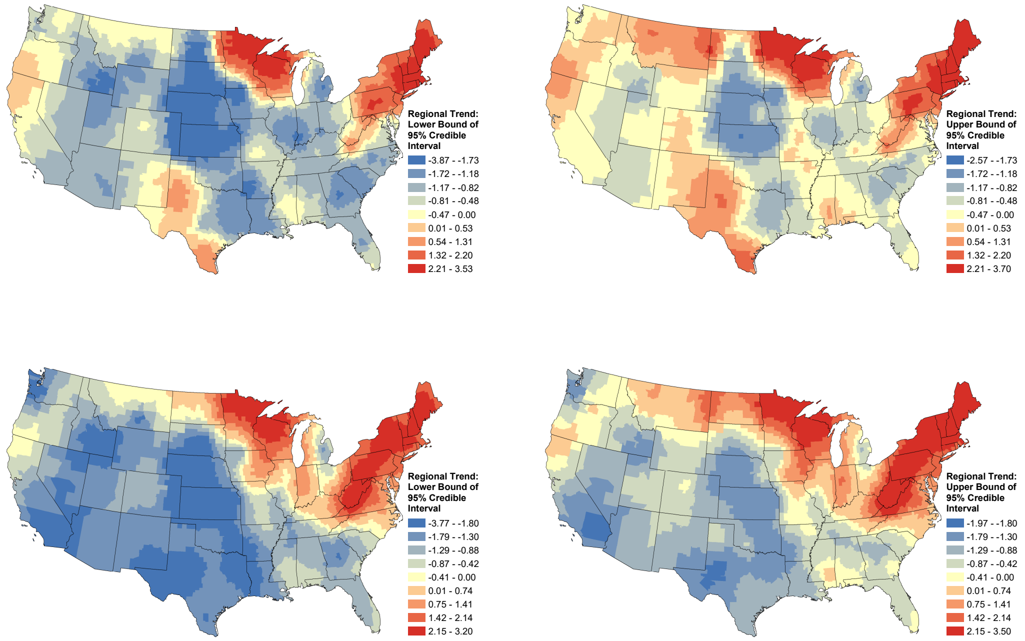

Web Figure 2: Lower (left) and upper (right) bounds of 95% credible intervals for the regional *Anaplasma* spp. (top) and *B. burgdorferi* (bottom) trends.

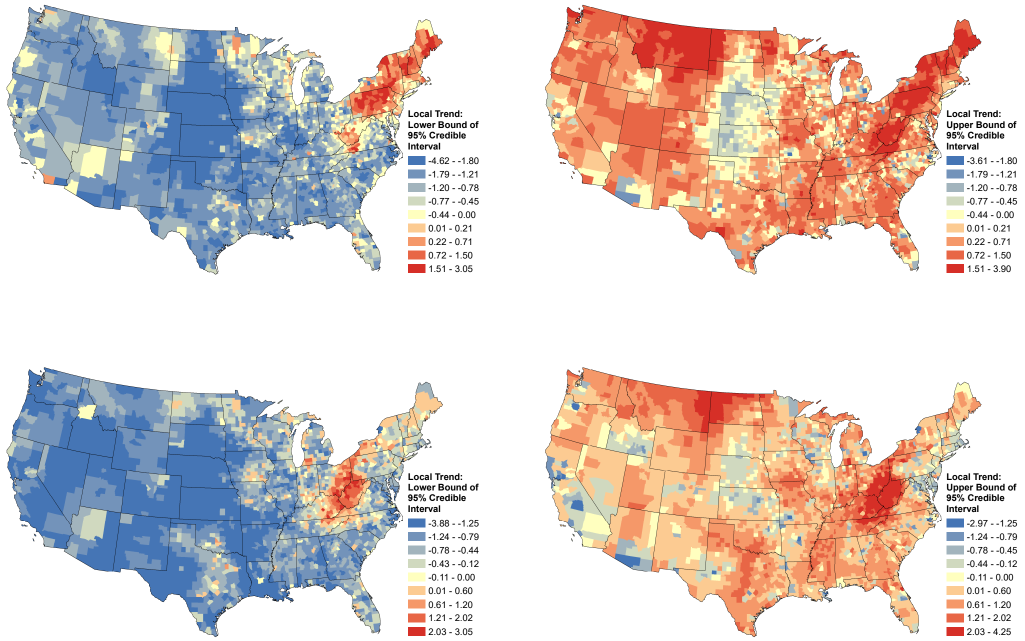

Web Figure 3: Lower (left) and upper (right) bounds of 95% credible intervals for the local *Anaplasma* spp. (top) and *B. burgdorferi* (bottom) trends.

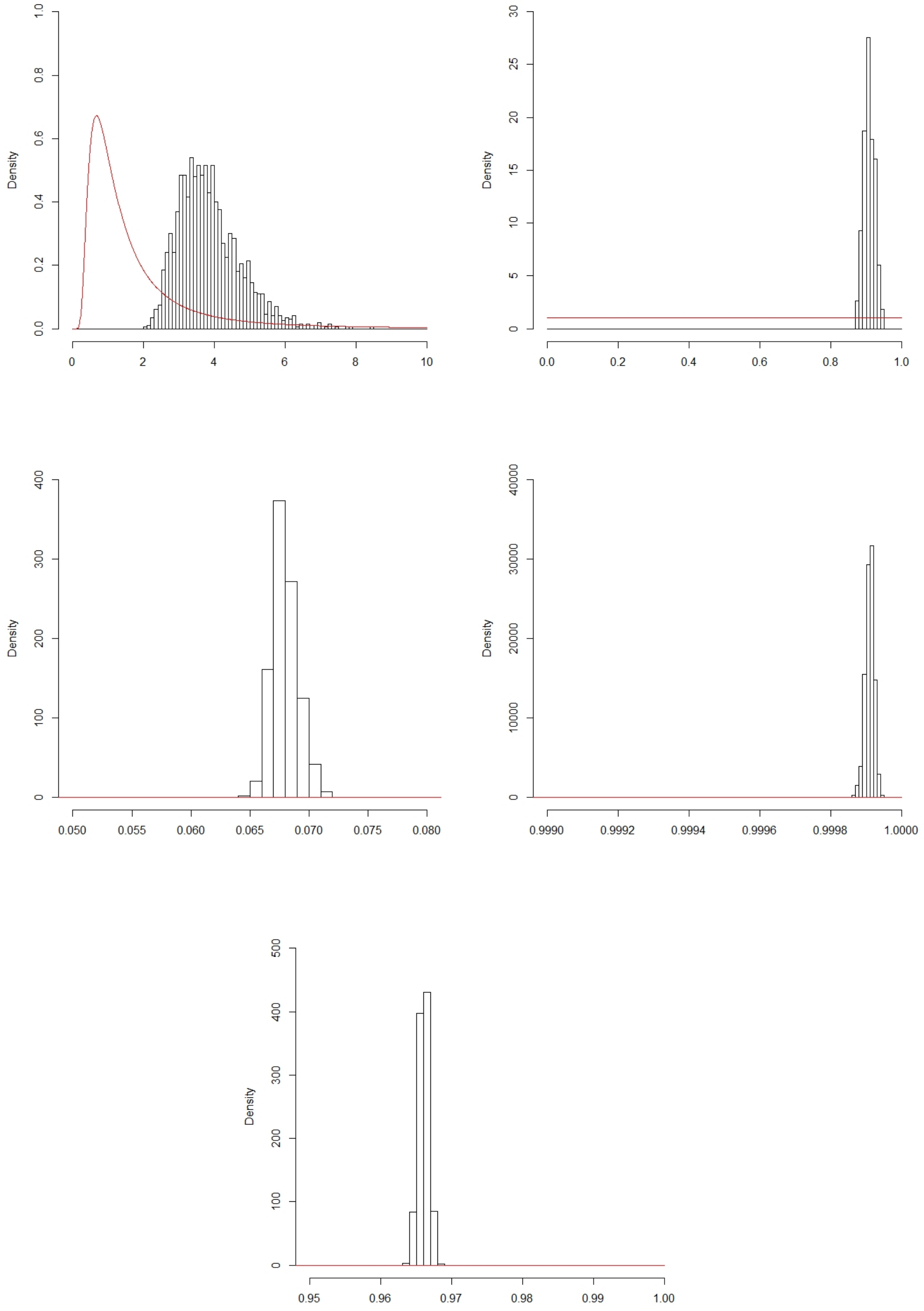

Web Figure 4: The figure displays the prior distribution (red) and posterior MCMC sample of the hyperparameters from the *B. burgdorferi* model. The plots corresponde to σ_1^2 (top left), θ_1 (top right), τ^2 (middle left), ω (middle right) and ζ (bottom).

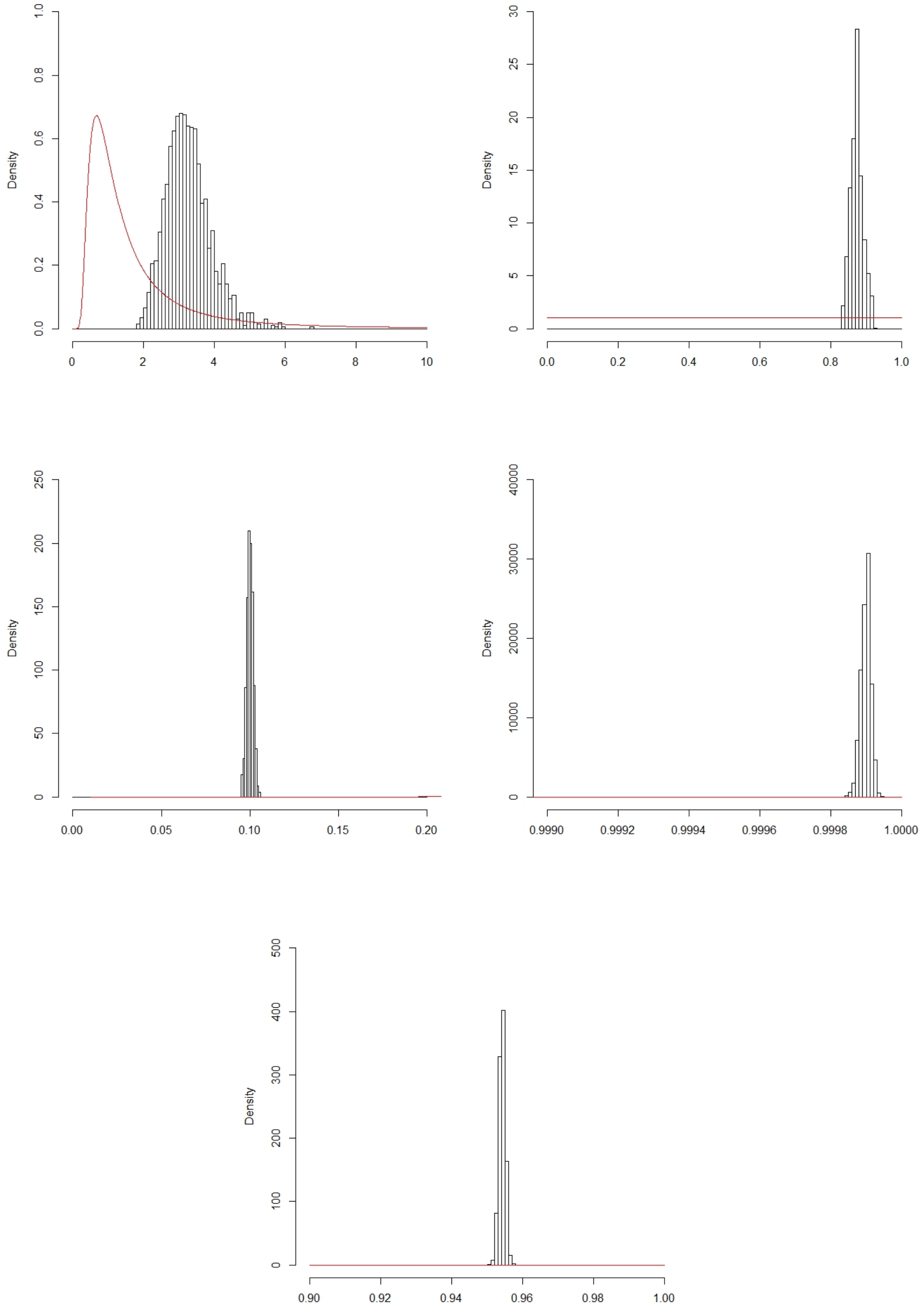

Web Figure 5: The figure displays the prior distribution (red) and posterior MCMC sample of the hyperparameters from the *Anaplasma* spp. model. The plots corresponds to σ_1^2 (top left), θ_1 (top right), τ^2 (middle left), ω (middle right) and ζ (bottom).

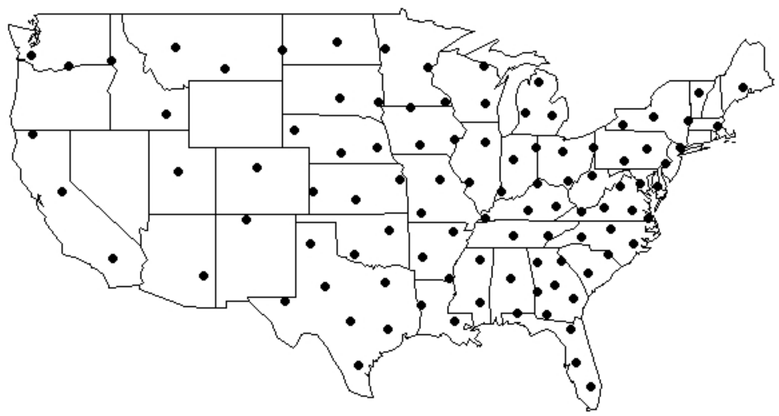

Web Figure 6: The figure displays the 100 knot locations used for the Gaussian predictive process.

References

Polson, N. G., Scott, J. G., and Windle, J. (2013). Bayesian inference for logistic models using pólya–gamma latent variables. *Journal of the American statistical Association*, 108(504):1339–1349.

Self, S. C. W., McMahan, C. S., Brown, D. A., Lund, R. B., Gettings, J. R., and Yabsley, M. J. (2018). A large-scale spatio-temporal binomial regression model for estimating seroprevalence trends. *Environmetrics*, 29(8):e2538.
